# Locally advanced basal cell carcinoma treated with sonidegib: *in vivo* monitoring with line-field confocal optical coherence tomography

**DOI:** 10.1093/skinhd/vzae025

**Published:** 2025-02-14

**Authors:** Simone Cappilli, Maria Mannino, Gerardo Palmisano, Enrico Bocchino, Alfredo Piccerillo, Andrea Paradisi, Alessandro Di Stefani, Ketty Peris

**Affiliations:** Dermatologia, Dipartimento di Medicina e Chirurgia Traslazionale, Università Cattolica del Sacro Cuore, Rome, Italy; UOC di Dermatologia, Dipartimento di Scienze Mediche e Chirurgiche, Fondazione Policlinico Universitario A. Gemelli-IRCCS, Rome, Italy; Dermatologia, Dipartimento di Medicina e Chirurgia Traslazionale, Università Cattolica del Sacro Cuore, Rome, Italy; UOC di Dermatologia, Dipartimento di Scienze Mediche e Chirurgiche, Fondazione Policlinico Universitario A. Gemelli-IRCCS, Rome, Italy; Dermatologia, Dipartimento di Medicina e Chirurgia Traslazionale, Università Cattolica del Sacro Cuore, Rome, Italy; UOC di Dermatologia, Dipartimento di Scienze Mediche e Chirurgiche, Fondazione Policlinico Universitario A. Gemelli-IRCCS, Rome, Italy; Dermatologia, Dipartimento di Medicina e Chirurgia Traslazionale, Università Cattolica del Sacro Cuore, Rome, Italy; UOC di Dermatologia, Dipartimento di Scienze Mediche e Chirurgiche, Fondazione Policlinico Universitario A. Gemelli-IRCCS, Rome, Italy; Dermatologia, Dipartimento di Medicina e Chirurgia Traslazionale, Università Cattolica del Sacro Cuore, Rome, Italy; UOC di Dermatologia, Dipartimento di Scienze Mediche e Chirurgiche, Fondazione Policlinico Universitario A. Gemelli-IRCCS, Rome, Italy; Dermatologia, Dipartimento di Medicina e Chirurgia Traslazionale, Università Cattolica del Sacro Cuore, Rome, Italy; UOC di Dermatologia, Dipartimento di Scienze Mediche e Chirurgiche, Fondazione Policlinico Universitario A. Gemelli-IRCCS, Rome, Italy; Dermatologia, Dipartimento di Medicina e Chirurgia Traslazionale, Università Cattolica del Sacro Cuore, Rome, Italy; UOC di Dermatologia, Dipartimento di Scienze Mediche e Chirurgiche, Fondazione Policlinico Universitario A. Gemelli-IRCCS, Rome, Italy; Dermatologia, Dipartimento di Medicina e Chirurgia Traslazionale, Università Cattolica del Sacro Cuore, Rome, Italy; UOC di Dermatologia, Dipartimento di Scienze Mediche e Chirurgiche, Fondazione Policlinico Universitario A. Gemelli-IRCCS, Rome, Italy

## Abstract

**Background:**

Hedgehog pathway inhibitors, including sonidegib and vismodegib, represent the treatment strategy for ‘difficult-to-treat’ basal cell carcinoma (BCC), encompassing, among others, locally advanced (laBCC) and metastatic BCC. Assessment of therapy response is challenging due to the presence of telangiectasia and scar tissue at the area of the BCC lesion. Line-field confocal optical coherence tomography (LC-OCT) is a new noninvasive imaging technique that provides high-resolution visualization of skin structures.

**Objectives:**

To investigate the value of LC-OCT for the assessment of laBCC response to sonidegib therapy.

**Methods:**

We retrospectively included patients with laBCC treated with sonidegib in the period from May 2020 to May 2023. Patients with laBCC underwent LC-OCT at baseline before starting sonidegib, and after sonidegib discontinuation when clinical complete response (CR) was reached. A subset of patients underwent LC-OCT assessment during sonidegib therapy to assess tumour persistence.

**Results:**

Twenty laBCCs in 20 patients [4 women, 16 men; mean (SD) age 76 (18) years] treated with oral sonidegib 200 mg daily were included in the study. Ten patients obtained an apparent clinical CR; LC-OCT imaging confirmed CR in 7/10 patients (70%), while in the remaining patients (3/10, 30%) LC-OCT revealed findings indicative of BCC non-CR. Ten patients were continuing sonidegib treatment: in this group LC-OCT revealed findings suggestive of BCC persistence in all 10 patients (100%).

**Conclusions:**

In this study we provide preliminary results of the beneficial use of LC-OCT in the management of patients with laBCC treated with sonidegib therapy.

## Teaser

Line-field confocal optical coherence tomography is a new non-invasive imaging technique that proved to be useful in the longitudinal monitoring of advanced basal cell carcinoma on sonidegib therapy.

**What is already known about this topic?**

- Assessment of locally advanced basal cell carcinoma (laBCC) response to sonidegib is challenging, and dermatoscopy is a useful tool for evaluation of complete clinical response and for tumour persistence.

**What does this study add?**

- Line-field confocal optical coherence tomography may be an additional, effective, noninvasive technique for the assessment of laBCC response to sonidegib.

Basal cell carcinoma (BCC) is the most common malignancy in white populations.^1^ The European Association of Dermato-Oncology (EADO) recently classified BCCs as ‘easy-to-treat’ and ‘difficult-to-treat’ according to patient and tumour characteristics.^2^ ‘Easy-to treat’ BCCs are commonly managed by standard surgery, radiotherapy or a variety of topical and destructive treatments; ‘difficult-to-treat’ BCCs, including, among others, locally advanced (laBCC) and metastatic BCC, are usually not eligible to surgery and/or radiotherapy and should be discussed in multidisciplinary meetings.^1,3^ Hedgehog pathway inhibitors (HHIs), including vismodegib or sonidegib, should be proposed for ‘difficult-to-treat’ BCCs, as the phase II ERIVANCE trial demonstrated a 39-month objective response rate (ORR) of 60.3% for patients with laBCC treated with vismodegib, whereas the phase II BOLT trial demonstrated a 42-month ORR of 56% for patients with laBCC on sonidegib.^4–7^

Real-life evaluation of HHI effectiveness can be difficult to perform due to the presence of scar tissue and telangiectasia in the area of the BCC lesion, and although dermatoscopy is useful for the noninvasive assessment of HHI therapy response, a biopsy is often required.^8^ Line-field confocal optical coherence tomography (LC-OCT) is an optical technique, introduced in 2018, that provides high-­resolution visualization of the different skin structures with a multimodal view [vertical, horizontal and three-dimensional (3D)], as it combines the principles of both reflectance confocal microscopy (RCM) and OCT.^9^ Preliminary results support its clinical value in the diagnosis of skin cancers, inflammatory conditions and cutaneous infectious diseases, with widening applications in numerous fields of clinical dermatology.^10^

The aim of the present study was to investigate the role of LC-OCT for the assessment of laBCC response to sonidegib therapy.

## Patients and methods

We retrospectively included patients with laBCC treated with sonidegib (200 mg daily) in the period from May 2020 to May 2023 at Fondazione Policlinico Universitario Agostino Gemelli IRCCS Rome, Italy. Patients with laBCC underwent LC-OCT at baseline before starting sonidegib, and after sonidegib discontinuation when apparent clinical complete response (CR) (referring to clinical and dermoscopic assessment) was reached. A subset of patients underwent LC-OCT evaluation during sonidegib therapy to assess for tumour persistence.

LC-OCT (CE-marked DeepLive, DAMAE Medical^®^, Paris, France) is a noninvasive device that acquires bidimensional (vertical and horizontal) images/videos in grey scale up to superficial/mid-dermis (depth acquisition ∼500 µm) with high-resolution (∼1.3-µm lateral resolution, ∼1.1-µm axial resolution). The software can also generate instant 3D cubes/slices in specific areas of high interest for the examiner.^9^

For patients who achieved a clinical CR, LC-OCT was performed on the area of the previously treated laBCC; for patients who continued sonidegib therapy due to non-apparent clinical CR achievement, the exam was performed on selected areas judged suspicious for disease persistence based on clinical–dermatoscopic features. Digital clinical and dermoscopic images (Dermalview Dual, Gavimedica, Camposano, Italy) were collected at baseline and at each scheduled follow-up for each patient.

## Results

Twenty laBCCs in 20 patients [4 women, 16 men; mean (SD) age 76 (18) years] treated with oral sonidegib were included in the study. Tumours were located on the face (14/20, 70%), on the trunk (3/20, 15%) and on the lower limbs (3/20, 15%). Anatomical areas of the face included eyelids (6/14, 30%), nose (5/14, 25%), auricular region (2/14, 10%) and scalp (1/14, 5%). LC-OCT was performed in all patients at baseline prior to starting therapy; then LC-OCT was repeated in 10 patients showing an apparent clinical CR, and in 10 patients continuing sonidegib treatment.

**Figure 1:**
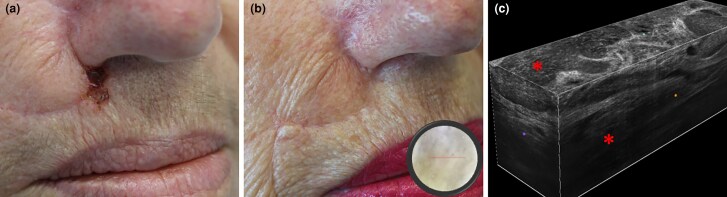
Clinical aspect and LC-OCT of laBCC in the right nasolabial fold: (a) recurrence of ulcerated tumour at the site of previous surgical excision, (b) showing an apparent clinical and dermatoscopic complete response after 13 months of sonidegib therapy; (c) three-dimensional LC-OCT image showing bright ovoidal dermal lobules (asterisks) corresponding to basaloid islands. laBCC, locally advanced basal cell carcinoma; LC-OCT, line-field confocal optical coherence tomography.

**Figure 2:**
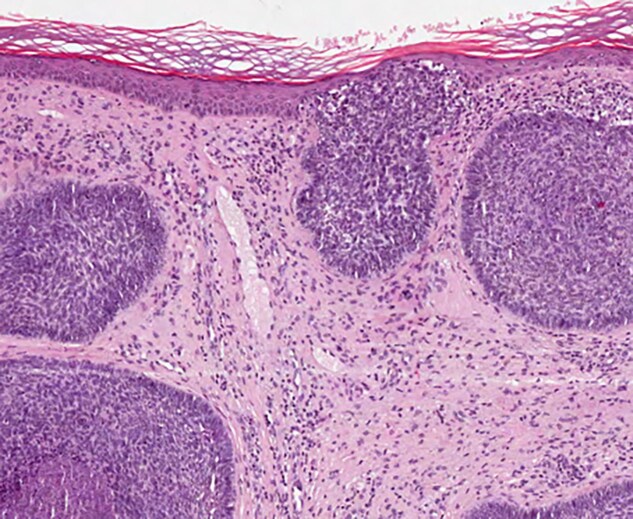
Histopatology of nodular basal cell carcinoma (staining haematoxylin and eosin, ×100).

Ten patients obtained an apparent clinical CR, with a mean duration of therapy of 9.5 months (range 4–13). LC-OCT was performed on the eyelid area (*n* = 4), nose (*n* = 3), auricular area (*n* = 1) and scapular area (*n* = 2). Imaging confirmed CR in 7/10 patients (70%), while in the remaining cases (3/10, 30%) LC-OCT revealed findings indicative of BCC persistence (Table 1). Patients with no signs of BCC were regularly monitored with clinical–dermoscopic exam, with no recurrence observed at the 24-month follow-up. Patients with LC-OCT imaging indicative of BCC non-CR were scheduled for surgical excision, and in all cases (*n* = 3) the histopathological report confirmed the diagnosis of BCC (*n* = 1 nodular BCC, *n* = 2 micronodular BCCs) (Figures 1 and 2).

**Table 1:** Clinical strategies for laBCC based on LC-OCT digital imaging

	Mean duration of therapy	LC-OCT findings	Clinical approach
Sonidegib therapy completed (10 patients)	9.5 months (range 4–13)		
7 patients		BCC complete response	Follow-up (no recurrence after 24 months)
3 patients		BCC persistence	Surgery
Sonidegib therapy continued (10 patients)	14.9 months (range 6–29)		
10 patients		BCC persistence	6 patients, sonidegib; 2 patients, surgery; 1 patient, radiotherapy; 1 patient, IMQ 5%

IMQ, imiquimod; laBCC, locally advanced basal cell carcinoma; LC-OCT, line-field confocal optical coherence tomography.

**Figure 3:**
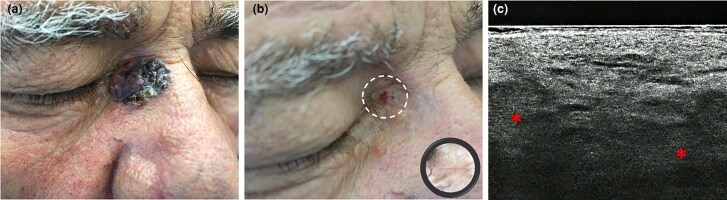
Clinical aspect and LC-OCT of laBCC in the medial canthus of the right eye: (a) large ill-defined pigmented plaque, (b) showing a residual focal erosion during therapy with sonidegib (12 months), with dermatoscopic features of telangiectasis and superficial crusting (inset); (c) vertical LC-OCT revealing bright ovoidal dermal structures (asterisks) as strictly related to basaloid islands. laBCC, locally advanced basal cell carcinoma; LC-OCT, line-field confocal optical coherence tomography.

**Figure 4:**
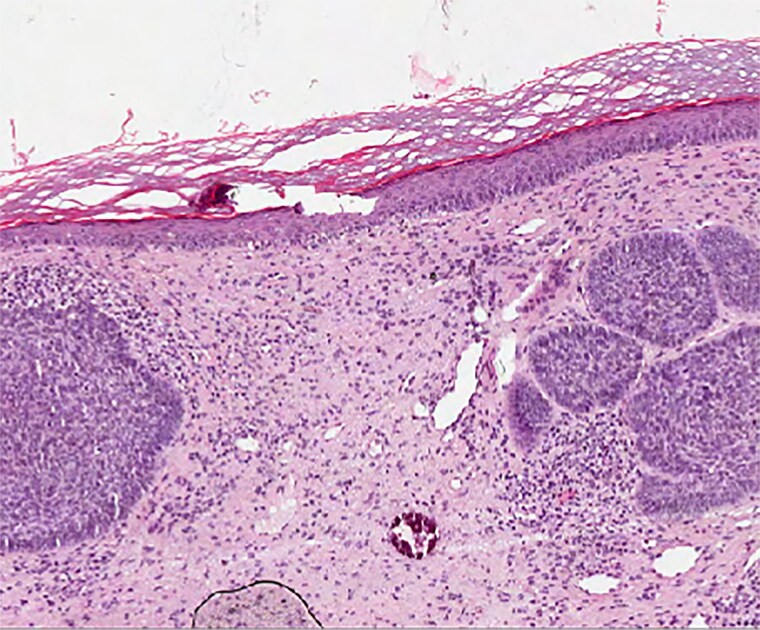
Histopatology of nodular basal cell carcinoma (staining haematoxylin and eosin, ×100).

Ten patients were continuing sonidegib treatment, and they had a mean duration therapy of 14.9 months (range 6–29). Target areas investigated by LC-OCT involved eyelid (*n* = 2), nose (*n* = 2), lower limbs (*n* = 3), trunk (*n* = 1), auricular area (*n* = 1) and scalp (*n* = 1). In this group, LC-OCT revealed findings suggestive of BCC persistence in all 10 patients (100%) under treatment. Different treatment strategies were adopted: 6 patients continued sonidegib, 2 patients underwent radical surgery after margin delineation by LC-OCT, resulting in complete excision (Figures 3 and 4); 1 patient was treated with radiotherapy with no clinical recurrence at 6-month follow-up; 1 patient was treated with imiquimod 5% cream (5 times weekly for 6 weeks) with no clinical recurrence at the 12-month follow-up (Table 1).

## Discussion

New emerging technologies have widened the armamentarium of the diagnostic skin imaging techniques available in clinical setting. In this study we provide the preliminary results of the beneficial use of LC-OCT in the management of patients with laBCC treated with systemic therapy. Subclinical BCC non-CR after interruption of sonidegib was approached mainly with surgery, drawing advantages from LC-OCT for the delineation of peripheral margins, as its application allowed a more precise and efficient surgical planning with smaller size defects.^11^ The histopathological report was concordant with LC-OCT in all cases, confirming its reliability. The persistence of BCC-related findings during treatment led to continuing therapy with sonidegib, and to considering radical surgery whenever feasible, or a switch to topical therapy (imiquimod 5%) or radiotherapy. Conversely, the confirmation of BCC clinical CR allowed us to schedule regular clinical monitoring in patients who had already withdrawn the drug. Previous studies have highlighted the advantages of digital imaging (RCM and dynamic OCT) over clinical exams and dermatoscopy for the outcome evaluation of BCC systemic therapy.^12,13^ In the near future, digital imaging may replace incisional biopsy, avoiding functional or major aesthetic sequelae in patients with a complex clinical scenario. Indeed, LC-OCT – providing practical key clues on disease activity – may be of added value in the monitoring of laBCC treated with systemic therapy, actively influencing clinical decision-making.

## Data Availability

The data underlying this article will be shared on reasonable request to the corresponding author.
